# DNA Circuits That Learn: Biochemical Signal Processing for Personalized Diagnosis and Treatment

**DOI:** 10.1002/mco2.70573

**Published:** 2025-12-29

**Authors:** Wen Yan Huang, Kyunghee Noh

**Affiliations:** ^1^ Bionanotechnology Research Center Korea Research Institute of Bioscience and Biotechnology (KRIBB) Yuseong‐gu Daejeon Republic of Korea; ^2^ Department of Nanobiotechnology KRIBB School of Biotechnology, UST Yuseong‐gu Daejeon Republic of Korea

1

In a recent *Nature* publication, Cherry and Qian [1] demonstrated a deoxyribonucleic acid (DNA)‐implemented neural network that performed supervised learning entirely in vitro via enzyme‐free, toehold‐mediated strand displacement. Class information was encoded as quantitative concentration states, which were reused to classify novel inputs within a single tube. This advancement embeds the concept of “learning” into chemistry itself, framing a retrainable, point‐of‐care decision layer.

Contemporary biomedicine is converging toward a hybrid paradigm that integrates artificial intelligence (AI)‐based foundation models, single‐cell and spatial transcriptomics, and biochemical/physical domain machine learning. However, practical constraints remain at the point of care. Currently deployed models are typically parameter fixed. Sensing, learning, and actuation are often decoupled in operations, while governance standards for foundation‐scale systems are still evolving. Under these circumstances, DNA neural network (DNA‐NN) ha s emerged as a chemistry‐native complement capable of co‐locating “learning” with measurement and actuation. These properties are naturally integrated with targeted‐therapy workflows, where near‐sample retraining and portable readouts can inform prognosis and treatment selection.

Historically, strand displacement circuitry—particularly the seesaw‐gate architecture—matured into winner‐take‐all classifiers that performed summation and thresholding at scale but relied on preinstalled weights (2011–2018) [2, 3]. In contrast, the 2025 study by Cherry and Qian represented a key inflection point. A one‐tube DNA‐NN executed supervised learning by writing labeled examples into concentration‐encoded memories, converting those memories into weights, and reusing them to classify novel inputs, all while maintaining independence, integration, generality, and stability. Taken together, these steps shifted molecular signal processing from fixed logic to adaptive logic.

Mechanistically, training inputs paired with labels drove the toehold‐mediated strand displacement (Figure 1), which accumulated class‐specific activators. These concentration memories were then transformed into weight‐bearing complexes. During inference, the presentation of a test pattern triggered bitwise interactions with these weights, and competitive nonlinearity yielded a winner readout, typically detected by fluorescence. As training, memory formation, and inference occur under isothermal, enzyme‐free conditions within a single reaction, the same network can be reconditioned with a newly labeled material without redesign. This operational property was directly relevant to workflows at the patients’ bedside [1].

**FIGURE 1:**
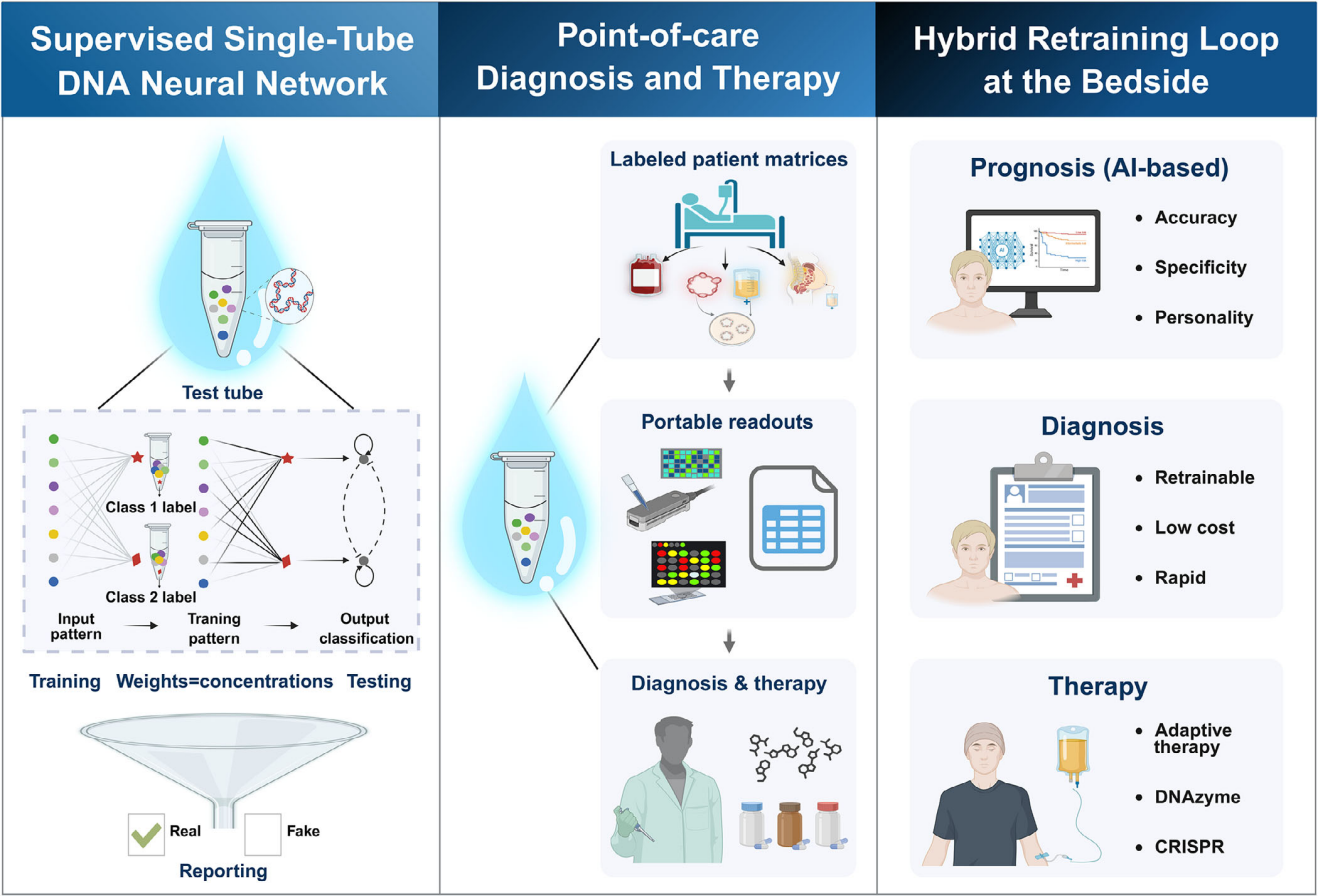
DNA‐implemented neural network (DNA‐NN) performs supervised learning through enzyme‐free, toehold‐mediated strand displacement, encoding class labels as molecular concentration states that can be retrained within a single tube. These properties align with targeted‐therapy workflows, where point‐of‐care retraining and portable readouts (e.g., nanopore or microfluidic assays) may inform prognosis and guide treatment selection. In principle, DNA‐NN chemistry can also support colocated sensing and adaptive reconditioning, and actuate downstream effectors such as DNAzymes, CRISPR‐associated modules, or prodrug‐activation circuits to enable closed‐loop prognosis‐to‐therapy. With foundation‐scale AI priors, single‐cell or spatial feature anchoring, and benchmarking of speed, specificity, and stability, DNA‐NN may be positioned as a retrainable, patient‐personalized point‐of‐care decision layer. Figure created with BioRender.com.

Many countries are now progressively reducing the use of animal experiments—which were once mandatory in drug development—and are accelerating the adoption of patient‐derived organoids (PDOs) as in vitro testbeds for preclinical assessment and mechanism‐aware stratification. In this context, the isothermal operation and retrainability of DNA‐NN is particularly attractive. A practical workflow is “training‐on‐sample”: (i) reconditioning the circuit using labeled PDOs supernatants or matched clinical matrices, such as plasma, synovial fluid, or pleural effusion; (ii) preservation of the resulting concentration‐encoded weights; and (iii) scoring of the follow‐up specimens using portable fluorescence or nanopore readouts at the point‐of‐care to support risk stratification and prognosis [1, 4].

Although the study is rooted in molecular computing, its implications extend directly to biomedicine. Smart diagnostics may involve DNA circuits that not only detect disease biomarkers but also learn from prior encounters, thereby improving sensitivity and specificity over time. Adaptive therapeutics could be realized using programmable DNA‐based drugs that refine responses with experience, akin to immune memory. Synthetic biology and precision medicine could integrate learning modules into artificial cells or therapeutic circuits, enabling dynamic adaptation to tumor evolution, immune evasion, or drug resistance. The study signals a shift from static molecular designs toward evolving therapeutic intelligence at the nanoscale. Importantly, this approach could be extended beyond diagnostics to therapy‐related applications, such as adaptive drug release systems or closed‐loop theragnostic platforms. For example, a DNA‐NN trained to recognize biomarker patterns associated with drug responsiveness could control downstream modules such as DNAzymes, CRISPR‐associated effectors, or prodrug activation circuits to enable precision‐targeted therapeutic action.

Despite these opportunities, certain important constraints remain. Current DNA‐NN operates on minute‐to‐hour kinetics dictated by strand displacement and diffusion. Scaling to multiple classes or longer patterns increases the crosstalk and leakage. Moreover, overcoming current limitations in scalability and reusability is essential, as DNA learning consumes energy and its circuits are currently “use‐once” only. Robust negative weights and stronger nonlinearities are still under development, and memory stability can be challenged under nuclease‐rich conditions. Moreover, readouts face trade‐offs between multiplexing and dynamic range. Nevertheless, mitigation strategies are still emerging. The 2025 *Nature* study employed clamp/cleanup strands together with tuned annealing stoichiometry to suppress spurious displacement and sharpen competition; additional tutorials in the study consolidated orthogonality design rules, leak control, and nanopore readouts specifically relevant to training‐on‐sample workflows [1, 4]. Future advances should focus on extending beyond supervised to unsupervised learning, enabling autonomous adaptation without external labels. Applying spatially organized DNA condensates or reaction–diffusion systems may allow scaling of network complexity. Microfluidic compartmentalization and nuclease‐resistant backbones are expected to stabilize memory and improve specificity at this scale.

Thus, DNA‐NN could provide a route toward colocated sensing, learning, and molecular actuation. Because knowledge is stored as concentrations rather than as immutable sequences, circuits can be reconditioned near the sample, at least in principle. After staged validation in PDOs and other microphysiological systems, these networks could be coupled with therapeutic modules to achieve closed‐loop control for prognosis and treatment via DNAzymes or clustered regularly interspaced short palindromic repeats (CRISPR)‐associated payloads [1, 4]. Furthermore, programmable DNA molecular networks are now expanding from discrete classification to continuous system tasks, further broadening the computational headroom within the same chemistry [5]. Together, these advances position DNA‐based neural network as a new paradigm at the interface of computation, biology, and medicine, with a potential impact on signal transduction research and targeted therapy strategies. With ongoing progress in orthogonal sequence design, leak suppression, robust readouts including nanopore integration, and microfluidic/nuclease‐resistant implementations, the field appears prepared to deliver a retrainable point‐of‐care decision layer. A pragmatic path forward is to retain foundation‐scale AI for cohort‐level priors and scalable inferences, anchor features in single‐cell and spatial transcriptomics, and incrementally benchmark DNA‐NN for speed, specificity, and stability. Following this, learning biochemistry may evolve into a practical engine for prognostic stratification and targeted interventions at the patients’ bedside.

## Author Contributions

W.Y.H. drafted the manuscript and generated the figure. K.N. reviewed and supervised the final manuscript. Both authors have read and approved the final manuscript.

## Conflicts of Interest

None

## Funding

This work was supported by NRF grants funded by Korea government (RS‐2024‐00338397) and KRIBB Research Initiative Program (KGM1062511 and KGM1322511).

## Ethics Statement

The authors have nothing to report.

## Competing Interests

The authors declare no conflicts of interest.

## Data Availability

The authors have nothing to report.
